# Quantifying early pelvic radiation-induced bone injury with dual-energy CT: a rabbit model study of dynamic bone mineral and marrow adiposity changes

**DOI:** 10.3389/fmed.2026.1828144

**Published:** 2026-07-02

**Authors:** Haiping Liu, Chen Cheng, Jiamin Zhang, Mengwei Wu, Yuling Hu, Ling He, Zhengjun Li, Jinhua Wang

**Affiliations:** 1 Department of Radiology, Jiangxi Maternal and Child Health Hospital, Nanchang, Jiangxi, China; 2 Jiangxi Medical College, Nanchang University, Nanchang, Jiangxi, China; 3 Department of Radiology, Children’s Hospital of Chongqing Medical University, Chongqing, China; 4 Department of Radiology, Yichun Maternal and Child Health Hospital, Yichun, Jiangxi, China

**Keywords:** bone marrow adiposity, calcium quantification, dual-energy computed tomography, hydroxyapatite, pelvic, radiation-induced bone injury

## Abstract

**Purpose:**

Dual-energy computed tomography (DECT) has been applied to tumor radiotherapy planning but has rarely been used in post-treatment toxicity assessment. This study assesses the feasibility of using DECT to detect early pelvic radiation-induced bone injury (pRIBI).

**Methods:**

Thirty mature female New Zealand White rabbits were allocated at random into five distinct cohorts: one non-irradiated control group and four experimental groups subjected to a single 30-Gy radiation exposure, with euthanasia performed at 1, 2, 3, or 4 weeks following irradiation. All rabbits underwent DECT before and after radiotherapy and contents of calcium (Ca), hydroxyapatite (HAP) and fat at the seventh lumbar vertebra (L7) and femoral neck (FN) were measured. Postmortem quantitative analysis utilized hematoxylin and eosin (HE)-stained with adipocyte-area-ratio (%) to measure fat content, while Ca content was measured via muffle furnace. Statistical evaluation was conducted via one-way analysis of variance (ANOVA) accompanied by *post-hoc* comparisons, alongside Pearson correlation assessments. The DECT-derived Ca concentration was validated using a phantom study.

**Results:**

Phantom validation confirmed high DECT Ca quantification accuracy (*R*^2^ = 0.995). DECT revealed progressive demineralization at L7 and FN, with significant reductions in Ca and HAP (*P* < 0.05) following a triphasic trajectory: initial slow decline (weeks 0–1), accelerated loss (weeks 1–3), and late attenuation (weeks 3–4). Muffle furnace Ca measurements strongly correlated with DECT values (*r*^2^ = 0.949, *P* < 0.05). Fat peaked at week 1 (*P* < 0.05) and progressively declined thereafter, aligning with histopathology.

**Conclusion:**

DECT enables non-invasive detection of early pRIBI, characterized by Ca and HAP loss, and dynamic fat remodeling. These findings underscore its potential for unveiling early pRIBI.

## 1 Introduction

Cervical cancer is a significant health issue, being the fourth most prevalent cancer among women globally (1–3). Radiotherapy serves as a fundamental cornerstone in the therapeutic algorithm for all disease stages. In cases of early-stage malignancy (FIGO I-IIA), the National Comprehensive Cancer Network (NCCN) Guidelines (2024) indicate that definitive radiotherapy, which integrates external beam radiation therapy and brachytherapy, yields 5-year overall survival (OS) outcomes comparable to those of surgery, with a 5-year overall survival specifically ranging from 80 to 90% (4). In locally advanced cases (FIGO IIB-IVA), concurrent chemoradiotherapy remains the standard of care, offering a 5-year OS of 50–65% (5).

Nevertheless, radiotherapy induces collateral injury to healthy tissues alongside tumor cell ablation, precipitating complications including non-neoplastic bone pain, skeletal atrophy, and heightened vulnerability to pathological fractures (6). Importantly, emerging evidence indicates that pelvic fractures, encompassing insufficiency types, affect between 10 and 29% of cervical cancer cohorts undergoing radiotherapeutic intervention (7, 8). Moreover, the risk of hip and femoral neck fractures remains significantly elevated even 5 years post-treatment, with a hazard ratio of 1.66 reported for cervical cancer patients (9). These skeletal complications carry substantial clinical consequences, as pelvic fractures—particularly hip fractures—represent major a contributor to treatment-related morbidity and mortality. Alarmingly, unstable pelvic ring injuries are associated with mortality rates approaching 30% (10). Despite the established role of bone mineral density (BMD) as both a biomarker of osteoporosis and a strong predictor of fracture risk (11), it remains under-assessed in routine gynecologic oncology practice.

Early monitoring of BMD is crucial, as it enables clinicians to take intervention measures in advance to avoid or prevent the occurrence of fractures, thereby improving patient outcomes. Radiation-induced skeletal injury is defined by the depletion of functional osteoblasts, increased marrow adiposity, and microvascular compromise, collectively driving sustained bone loss alongside a transient expansion of bone marrow adipose tissue (BMAT) (12). Furthermore, emerging evidence highlights BMAT as a novel diagnostic and prognostic biomarker for bone quality (13, 14).

Although Dual-energy X-ray Absorptiometry (DXA) continues to serve as the important reference standard for quantifying bone mineral density (BMD), its failure to characterize marrow adiposity or microarchitectural alterations restricts its effectiveness in identifying early-stage radiation-induced bone injury (RIBI) (15). Quantitative magnetic resonance imaging (MRI) approaches, including water-fat imaging and proton magnetic resonance spectroscopy (1H-MRS), offer distinct benefits for evaluating bone marrow adipose tissue (BMAT) content and composition while avoiding ionizing radiation (16). Nevertheless, when evaluating trabecular bone architecture, magnetic resonance imaging (MRI) yields less comprehensive data than computed tomography (CT). Furthermore, MRI lacks the capacity to directly measure concentrations of calcium (Ca) and hydroxyapatite [HAP, Ca_10_(PO_4_)_6_(OH)_2_], the principal inorganic constituents within bone matrix (17). Standard computed tomography conflates the radiodensity of calcified structures with that of marrow elements, whereby elevated marrow adiposity compromises the precision of bone mineral density assessment.

As an innovative imaging modality, dual-energy CT (DECT) leverages the energy-dependent attenuation characteristics of X-rays in different materials to achieve superior tissue characterization and material decomposition capabilities (18). This technology has demonstrated particular promise in bone health assessment, enabling accurate BMD quantification through advanced material decomposition algorithms without requiring calibration phantoms (19). Clinical validation studies have confirmed the robustness of DECT-derived BMD measurements, showing excellent stability across varying radiation doses and strong correlation with quantitative CT (QCT) reference standards (20). A significant advantage of DECT lies in its unique potential for simultaneous evaluation of both BMD and BMAT content during a single acquisition. Importantly, DECT-quantified BMAT parameters show remarkable concordance with ^1^H-MRS measurements, the current non-invasive reference standard for fat quantification (21).

Although prior studies have utilized imaging modalities (MRI, DXA, CT) to quantify BMD changes following irradiation, direct correlative evidence bridging these imaging findings with definitive histopathology remains sparse. The rabbit serves as a pivotal experimental model for elucidating the complex interplay between bone cells and radiation therapy, offering superior insights into their biological interactions. Consequently, this investigation sought to assess the viability of DECT for characterizing pelvic RIBI, employing an animal model alongside muffle furnace assays and histomorphometric analysis as benchmark references.

## 2 Materials and methods

### 2.1 Phantom study

**FIGURE 1:**
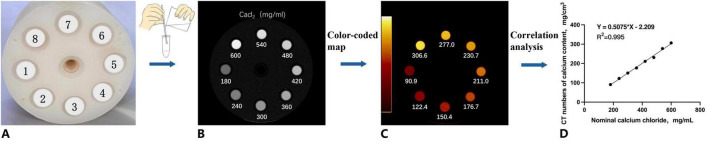
Schematic workflow of DECT phantom study. **(A)** Customized anthropomorphic phantom containing calcium chloride (CaCl_2_). **(B)** Conventional 70-kev image of phantom. **(C)** Color-coded calcium map of phantom. **(D)** Correlation analysis comparing measured vs. known concentrations (mg/mL) using region-of-interest (ROI) measurements. DECT, Dual-energy computed tomography.

Based on established methods for dipotassium phosphate (K_2_HPO_4_) /water phantom preparation (22), we prepared calcium chloride (Cacl_2_) solutions as follows: anhydrous Cacl_2_ (crystalline Cacl_2_, chemical grade, Shanghai Macklin Biochemical Technology Co., C805225) was dissolved in distilled water (3 g/5 mL) at room temperature to create a 100% saturated stock solution, which was then diluted to generate 30–90% gradient concentrations (in 10% increments). These solutions were loaded into eight polyvinyl chloride tubes (100 mm length, 25 mm outer diameter) inserted in a cylindrical plastic phantom, with each tube filled to 20 mL capacity and sealed. The DECT scanner (Revolution CT, GE HealthCare) was used to perform spectral imaging scans on the phantom, with the following scan protocol: fast kVp switching, 80/140 kVp; tube current, 370 mA; gantry rotation time, 0.6 s; helical pitch, 0.992:1; detector coverage, 80 mm; slice thickness, 5.0 mm; matrix size, 512 x 512. Three regions of interest (ROIs) of 150 mm^2^ was placed on three consecutive slices centrally in each tube. The measured mean Cacl_2_ concentrations were compared with the nominal values to assess accuracy and correlation (Figures 1A–C).

### 2.2 Animals and irradiation

Thirty adult female New Zealand White rabbits, aged 2–3 months with body weights ranging from 2.0 to 3.0 kg, were recruited from the Experimental Animal Center at Nanchang University. The rabbits were housed at the center under controlled environmental conditions: room temperature 21–24°C, relative humidity 50–70%, and a 12/12-h light/dark cycle. All rabbits received a standardized nutritional diet three times daily with *ad libitum*. Individual housing was provided in stainless steel cages with enrichment items. Following a 14-day acclimatization period with daily health monitoring, all subjects demonstrated normal physiological parameters and behavior. All protocols involving animal experimentation received formal authorization from the Committee for Ethical Use of Experimental Animals at Nanchang University (Approval ID: NCULAE-20250718001).

The 30 rabbits were randomly divided into five groups: normal control (no irradiation, 0-week), 1-, 2-, 3-, and 4-week after irradiation. Each group was clearly labeled on the rabbit cages. All rabbits in the experimental groups underwent a baseline DECT scan before irradiation. For this scan, animals were anesthetized with intravenous propofol (2–3 mg/kg) and positioned on the DECT scanner couch. Anesthesia depth was monitored by corneal reflex and respiratory rate. After completing the baseline DECT scan, the rabbits were immediately transferred to the linear accelerator room for irradiation while still under anesthesia. For irradiation, the same anesthesia was maintained. Rabbits were placed in the supine position on the linear accelerator couch. A single dose of 30 Gy was delivered to the pelvic region through the ventral approach using 6 MV X-ray beam (Varian 600-C^®^, Varian Medical Systems, Palo Alto, CA, United States). The beam was directed vertically from the ventral to the dorsal side (anteroposterior, AP). For radiation field localization, a test dose of 6 monitor units (MU) (approximately 0.06 Gy) was delivered with the rabbit on the couch for portal imaging verification. The total prescribed dose of 30 Gy was achieved by delivering a therapeutic dose of 29.94 Gy (2994 MU) following the test dose, ensuring that the animal received exactly 30 Gy including the test exposure. The radiation field covered the pelvic region, with the upper border of L7 vertebra superiorly, S2 vertebra inferiorly, and bilateral femoral necks (FNs) laterally. Radiation was delivered through a 15 × 15 cm^2^ at a source-to-skin distance (SSD) of 100 cm with a dose rate of 500 MU/min. After radiotherapy, the animals were allowed to recover naturally and were then returned to standard housing cages with free access to food and water. Weekly examinations were conducted to monitor and record the condition of the irradiated area, including hair, skin, weight, appetite and limb mobility.

**FIGURE 2:**
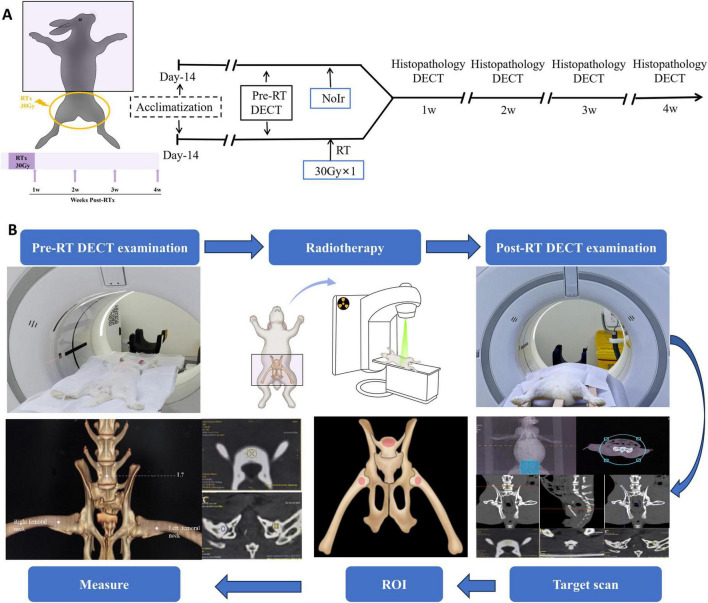
Radiotherapy response assessment in rabbits: time course of local irradiation and DECT workflow. **(A)** Time course of local irradiation experiment in rabbits. **(B)** Workflow of radiotherapy response assessment using DECT. RT, Radiotherapy; DECT, Dual-energy computed tomography.

Post-irradiation DECT scans were performed at the designated endpoint for each group (1-, 2-, 3-, and 4-week). At each endpoint, rabbits were anesthetized again, a follow-up DECT scan was performed, and immediately after the scan the animals were weighed, sacrificed by decapitation under deep anesthesia induced by intravenous propofol (2–3 mg/kg) and the L7 lumbar vertebrae and bilateral FNs were collected for pathological examination and Ca content analysis. The complete experimental timeline and sampling protocol are presented in Figure 2A.

### 2.3 DECT imaging technique, acquisition and processing of images

All rabbits were scanned using a 256-row multidetector spectral CT system (Revolution CT, GE Healthcare). Initially, a comprehensive whole-body scan was performed on 30 rabbits, followed by targeted scan with a FOV of 12 × 12 cm, which covered the pelvic cavity from the L7 vertebra to the femoral neck. The imaging protocol employed Gemstone Spectral Imaging (GSI) in KV mode, utilizing rapid kVp switching between 80 and 140 kVp. Additional acquisition settings included a tube current of 355 mA, a helical pitch of 0.516:1, a gantry rotation duration of 0.8 s, detector coverage spanning 40 mm, a reconstructed slice thickness of 0.625 mm, and an image matrix of 512 × 512.

All DECT datasets were exported to an Advantage Workstation (AW4.7; GE Healthcare) for subsequent processing, utilizing GSI software alongside the material decomposition (MD) algorithm. Two experienced musculoskeletal radiologists (each with 10 years of experience) delineated ROIs within the cancellous bone of L7 vertebrae and bilateral FNs on 70 keV virtual monoenergetic images (VMIs), carefully avoiding the central high-density zone (e.g., bone islands, calcifications, and vascular structures), areas within 5 mm of cortical margins, and imaging artifacts (e.g., beam-hardening artifacts, motion artifacts, and beam-related streak artifacts). To ensure precise spatial alignment between pre- and post-radiotherapy images, ROIs were aligned through a process guided by 3D reconstruction, standardized anatomical positioning, and self-comparison. These ROIs were subsequently propagated to Ca (Fat)-based MD images, HAP (Fat) -based MD images, and Fat (Ca)/Fat (HAP) maps for quantitative measurement of Ca, HAP, and fat content. Three consecutive measurements were obtained at each site and averaged.

Quantitative parameters derived from DECT material decomposition-including Ca (Fat), HAP (Fat), Fat (Ca), and Fat (HAP)-were expressed as post- to pre-radiotherapy ratios. These ratios were calculated by dividing the value obtained at each post-radiotherapy time point by the corresponding pre-radiotherapy (baseline) value from the same anatomical region. Relative values were calculated as the ratio of post-radiotherapy to baseline concentrations (Figure 2B). Subsequently, using the same method described above, Ca, HAP and fat content were measured in the non-irradiated region (L4 vertebra). Additionally, CT attenuation values of 70 keV VMIs were measured at the corresponding sites of the L7 vertebra and bilateral FNs, with the results expressed as ΔCT.

### 2.4 Histomorphometric analysis and calcium content detection

Following decapitation, L7 vertebrae and bilateral FNs were surgically extracted, cleared of soft tissue, and rinsed with saline. Samples were either snap-frozen in liquid nitrogen and stored at −80°C for calcium content analysis or fixed in 4% paraformaldehyde (PFA; 24 h at 4°C) for histological examination. The PFA-fixed specimens were decalcified in 10% nitric acid for 5−7 days with solution replacement every 48 h. Decalcification endpoint was determined by needle-puncture testing. Following paraffin embedding, 10-μm sections were prepared and subjected to hematoxylin and eosin (HE) staining. Adipocyte proportions in medullary regions were quantified using Image-Pro Plus software, with eight randomly selected fields (40 × magnification) analyzed per group.

Bone samples were ashed at 550°C for 4 h in a muffle furnace (KDF1500-plus, Yamato Scientific Company) until complete whitening. Exactly 0.5 g of resulting ash was dissolved in 10 mL 0.5% HNO_3_, diluted to 250 mL with ultrapure water, and homogenized. For analysis, sample were further diluted at a ratio of 1:10 and measured by flame atomic absorption spectrophotometer (SP-3590AA, Shanghai Spectrum Instruments Co., Ltd.) under optimized conditions: flame method of air-acetylene rich flame, analytical wavelength of 422.7 nm, lamp current of 10 mA, spectral bandwidth of 0.5 nm, and sample intake rate of 8 mL/min. Ca concentrations (mg/L) were determined against certified standard curves, with final bone Ca content (mg/g) calculated by normalizing to the initial ash weight.

### 2.5 Statistical analysis

All statistical computations were executed via SPSS version 25.0 (IBM Corp.). Continuous variables are reported as mean ± standard deviation (SD). Before applying parametric tests, we verified data normality using the Shapiro-Wilk test and assessed variance homogeneity with Levene’s test. We employed one-way analysis of variance (ANOVA) to examine post-radiotherapy body weight fluctuations, inter-group disparities in Ca (Fat), HAP (Fat), Fat (Ca), and Fat (HAP) levels, alongside ΔCT values. For *post-hoc* pairwise comparisons, we applied the LSD test when variances were homogeneous; otherwise, Tamhane’s T2 test was utilized. Pearson correlation coefficients quantified: (a) the relationship between spectral CT-derived calcium measurements and phantom CaCl_2_ concentrations, and (b) the concordance between spectral CT Ca (Fat) readings and muffle furnace Ca determinations (*r* > 0.700, indicating strong agreement). All graphical representations were generated using GraphPad Prism 9.0. A *P*-value < 0.05 defined statistical significance.

## 3 Results

### 3.1 Phantom study

The concentrations of the 180, 240, 300, 360, 420, 480, 540, and 600 mg/mL Cacl_2_ inserts were measured as 90.900 ± 0.775, 122.400 ± 1.140, 150.400 ± 0.711, 176.700 ± 1.065, 211.000 ± 2.389, 230.700 ± 1.511, 277.000 ± 0.480, and 306.600 ± 1.690 mg/cm^3^ of the corresponding Ca (Water) based MD images, respectively. The measured CaCl_2_ concentrations exhibited a robust linear association with the nominal values, characterized by a coefficient of determination (*R*^2^) of 0.995 and statistical significance at *P* < 0.01 (Figure 1D).

### 3.2 General observations

In this study, rabbits resumed normal feeding and drinking behaviors within 4 h post-irradiation. Following radiotherapy, the body weight showed a decline at 1, 2, 3, and 4 weeks, with mean reductions of 7.50, 13.52, 7.85, and 13.77%, respectively (all *P* > 0.05). Reduced appetite became apparent in most rabbits approximately 2 weeks post-irradiation, with food intake decreasing by approximately one-quarter (from ∼60 to ∼45 g/day). By weeks 3 and 4 post-irradiation, approximately half of the rabbits in the irradiated group developed moderate perineal edema, and approximately one-third exhibited mild radiation enteritis accompanied by watery stools.

### 3.3 Radiological and quantitative analysis of bone marrow changes following radiation

**FIGURE 3:**
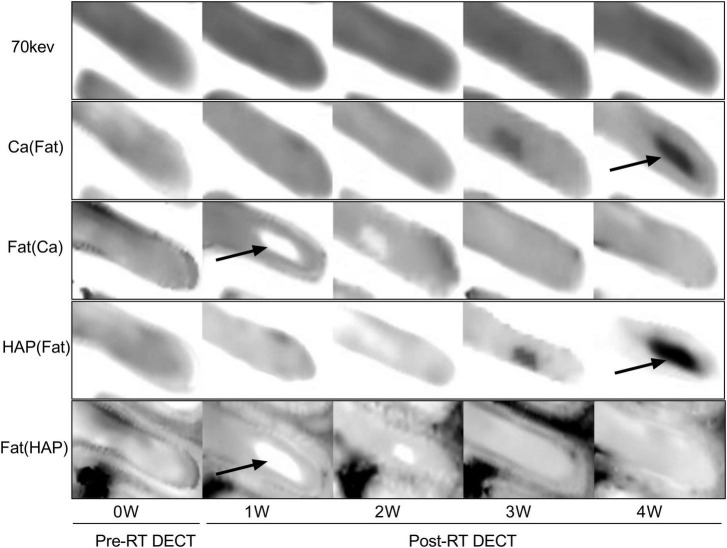
Imaging analysis of radiotherapy effects on the right femoral neck. Representative conventional single-energy CT (70 keV) images and corresponding DECT material decomposition maps: Ca-based MD images [Ca (Fat) and HAP (Fat)] and fat-based MD images [Fat (Ca) and Fat (HAP)] at different post-radiotherapy intervals.

Conventional 70-kev images revealed no structural changes in irradiated bone during week 1−4 (Figure 3). In contrast, the DECT material decomposition parameters showed distinct temporal evolution patterns. Representative images from the right FN demonstrated these dynamic changes. For Ca/HAP-based parameters [Ca (Fat) and HAP (Fat)], the signal intensity progressively decreased over time, with the lowest signal intensity (darkest area) in the bone marrow cavity observed at week 4, indicating minimal Ca/ HAP content at this time point. For fat-based parameters [Fat (Ca) and Fat (HAP)], the signal intensity transiently increased at week 1, exhibiting the highest signal intensity (brightest area), followed by a gradual decline in subsequent weeks.

DECT assessments of non-irradiated regions (L4 vertebra) showed no statistically significant differences in Ca, HAP, or fat content among the different post-irradiation interval groups (Table 1). The post- to pre-radiotherapy ratios for Ca (Fat), HAP (Fat), Fat (Ca), and Fat (HAP) in irradiated regions are summarized in Table 2.

**TABLE 1:** DECT quantification of bone composition in L4 vertebra at different post-radiotherapy intervals.

Region	After-therapy interval	Ca (Fat)	HAP (Fat)	Fat (Ca)	Fat (HAP)
	0 W	1.000 ± 0.000^a^	1.000 ± 0.000^a^	1.000 ± 0.000^a^	1.000 ± 0.000^a^
L4	1 W	0.977 ± 0.084^a^	1.109 ± 0.047^a^	1.006 ± 0.017^a^	1.002 ± 0.001^a^
	2 W	0.972 ± 0.100^a^	1.022 ± 0.107^a^	1.000 ± 0.003^a^	1.001 ± 0.003^a^
	3 W	1.248 ± 0.228^a^	1.716 ± 0.879^a^	1.779 ± 1.352^a^	1.772 ± 1.334^a^
	4 W	1.290 ± 0.308^a^	0.990 ± 0.107^a^	0.997 ± 0.003^a^	0.999 ± 0.005^a^
	*P*-value	*P* = 0.132	*P* = 0.204	*P* = 0.454	*P* = 0.449

Statistical significance among groups was assessed by one-way ANOVA followed by a *post-hoc* test (Duncana al significance a column, identical superscript letters (e.g., a) indicate no statistically significant difference (*P* ≥ 0.05).

**TABLE 2:** DECT measurement errors (mean ± SD) for Ca (Fat), HAP (Fat), Fat (Ca) and Fat (HAP).

Region	After-therapy interval	Ca (Fat)	HAP (Fat)	Fat (Ca)	Fat (HAP)	ΔCT
L7	0 W	1.000 ± 0.000^a^	1.000 ± 0.000^a^	1.000 ± 0.000^d^	1.000 ± 0.000^d^	−
	1 W	0.934 ± 0.015^b^	0.943 ± 0.024^b^	1.224 ± 0.029^a^	1.286 ± 0.021^a^	9.144 ± 3.772^a^
	2 W	0.822 ± 0.016^c^	0.827 ± 0.015^c^	1.127 ± 0.020^b^	1.149 ± 0.007^b^	24.789 ± 16.054^a^
	3 W	0.750 ± 0.016^d^	0.753 ± 0.015^d^	1.023 ± 0.010^c^	1.031 ± 0.007^c^	15.736 ± 6.551^a^
	4 W	0.687 ± 0.017^e^	0.686 ± 0.013^e^	0.991 ± 0.004^e^	0.995 ± 0.004^d^	13.910 ± 6.341^a^
	*P*-value	P < 0.001	P < 0.001	P < 0.001	P < 0.001	*P* = 0.299
Left femoral neck	0 W	1.000 ± 0.000^a^	1.000 ± 0.000^a^	1.000 ± 0.000^d^	1.000 ± 0.000^d^	–
	1 W	0.965 ± 0.021^a^	0.957 ± 0.029^b^	1.423 ± 0.009^a^	1.338 ± 0.008^a^	8.902 ± 5.639^b^
	2 W	0.855 ± 0.015^b^	0.857 ± 0.012^c^	1.135 ± 0.016^b^	1.261 ± 0.024^b^	18.316 ± 9.472^b^
	3 W	0.728 ± 0.006^c^	0.740 ± 0.007^d^	1.015 ± 0.005^c^	1.016 ± 0.007^c^	51.892 ± 31.425^a^
	4 W	0.675 ± 0.017^d^	0.699 ± 0.024^e^	0.989 ± 0.008^d^	0.989 ± 0.008^d^	28.925 ± 11.515^ab^
	*P*-value	*P* < 0.001	*P* < 0.001	*P* < 0.001	*P* < 0.001	*P* = 0.078
Right femoral neck	0 W	1.000 ± 0.000^a^	1.000 ± 0.000^a^	1.000 ± 0.000^d^	1.000 ± 0.000^cd^	–
	1 W	0.946 ± 0.017^b^	0.944 ± 0.015^b^	1.343 ± 0.024^a^	1.215 ± 0.014^a^	11.833 ± 5.316^a^
	2 W	0.831 ± 0.011^c^	0.852 ± 0.011^c^	1.138 ± 0.022^b^	1.142 ± 0.027^b^	20.183 ± 15.335^a^
	3 W	0.706 ± 0.004^d^	0.722 ± 0.018^d^	1.014 ± 0.003^c^	1.021 ± 0.013^c^	40.936 ± 21.180^a^
	4 W	0.688 ± 0.007^e^	0.684 ± 0.007^e^	0.981 ± 0.005^e^	0.983 ± 0.011^d^	36.263 ± 30.782^a^
	*P*-value	*P* < 0.001	*P* < 0.001	*P* < 0.001	*P* < 0.001	*P* = 0.328

Statistical significance among groups was assessed by one-way ANOVA followed by a *post-hoc* test (Duncan’s test). Different superscript letters (e.g., a, b) within a column denote statistically significant differences (*P* < 0.05), while means with the same letter are not significantly different (*P* ≥ 0.05).

**FIGURE 4:**
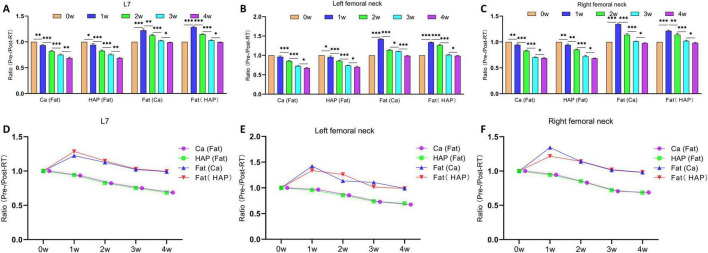
DECT quantified temporal changes during radiotherapy. **(A–C)** Bar graph and **(D–F)** line graph as displayed the DECT quantified temporal changes. All y-axes represent normalized values, calculated as the ratio of post-radiotherapy to pre-radiotherapy (baseline) concentrations for each material basis pair. Results are expressed as mean ± standard deviation (*P < 0.05, **P < 0.01, ***P < 0.001) (one-way analysis of variance followed by Tukey’s *post-hoc* test). DECT, Dual-energy computed tomography.

GSI measurements revealed progressive declines in bone mineralization indices [Ca (Fat) and HAP (Fat)] across all examined regions (L7 vertebra, bilateral FNs) over time post-irradiation (Figure 4). The decline was modest during week 1 [Ca (Fat): L7 6.6%, left FN 3.5%, right FN 5.4%; HAP (Fat): L7 5.7%, left FN 4.3%, right FN 5.6%], accelerated during weeks 2 [Ca (Fat): L7 12.0%, left FN 11.4%, right FN 12.2%; HAP (Fat): L7 12.3%, left FN 10.4%, right FN 9.7%] or 3 [Ca (Fat): L7 8.8%, left FN 14.9%, right FN 15.0%; HAP (Fat): L7 8.9%, left FN 13.7%, right FN 15.3%], and then decelerated by week 4 [Ca (Fat): L7 8.4%, left FN 7.3%, right FN 2.5%; HAP (Fat): L7 8.9%, left FN 5.5%, right FN 5.3%] (Figure 4).

In contrast, fat-based parameters [Fat (Ca) and Fat (HAP)] exhibited an initial increase at week 1, peaking at this time point [L7: Fat (Ca) 1.224 ± 0.029, Fat (HAP) 1.286 ± 0.021; left FN: 1.423 ± 0.009, 1.338 ± 0.008; right FN: 1.343 ± 0.024, 1.215 ± 0.014], representing relative increases of +22.4% to +42.3% for Fat (Ca) and +21.5% to +33.8% for Fat (HAP). Subsequently, these values gradually declined, reaching slightly below baseline levels by week 4 (Fat (Ca): L7 L)F%, left FNbseq%, right FNsequ%; Fat (HAP): L7 LP)%, left FNNseq%, right FNsequ%) (Figure 4).

Statistical comparisons (Duncan’s test) are detailed in Figure 4. For the L7 vertebra, significant temporal differences (*P* < 0.05) were observed for Ca (Fat), HAP (Fat), and Fat (Ca) among all time points, and for Fat (HAP) between most time points except week 0 vs. 4 (*P* = 0.391) (Figures 4A,D). In the left FN, HAP (Fat) differed significantly among all groups (*P* < 0.05); Ca (Fat) showed significant differences except week 0 vs. 1 (*P* = 0.095). Fat (Ca) and Fat (HAP) changes were significant except week 0 vs. 4 (*P* = 0.201/ 0.152) (Figures 4B,E). In the right FN, Ca (Fat), HAP (Fat), and Fat (Ca) all showed significant differences (*P* < 0.05) among all groups; Fat (HAP) was significant except week 0 vs. 3/4 (*P* = 0.090/ 0.133) (Figures 4C,F). No statistically significant differences in ΔCT values were observed among groups for any region.

### 3.4 Histological observations and calcium content detection

**FIGURE 5:**
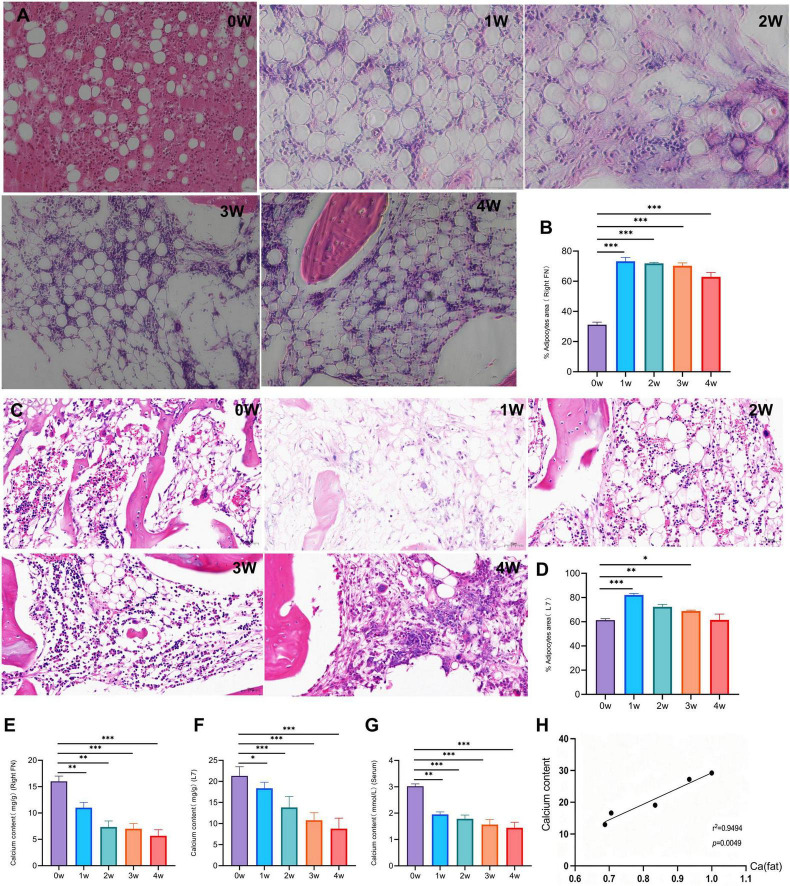
Histological analysis and correlation with calcium deposition assessed by DECT. **(A)** Representative HE staining of FN at different time points. Scale bar = 50 μm. **(B)** Quantification of the percentage adipocyte area (%) over the experimental period in the FN. **(C)** Representative HE staining of L7 at different time points. Scale bar = 50 μm. **(D)** Quantification of the percentage adipocyte area (%) over the experimental period in the L7. **(E–G)** Calcium content was measured. **(H)** Pearson correlation between DECT-derived Ca (Fat) values and muffle furnace-assayed calcium content in the right FN. Results are expressed as mean ± standard deviation (*P < 0.05, **P < 0.01, ***P < 0.001) (one-way analysis of variance followed by Tukey’s *post-hoc* test). DECT, Dual-energy computed tomography; HE, Hematoxylin and Eosin; FN, femoral neck.

Prior to irradiation, the bone marrow exhibited active proliferation of hematopoietic tissue, with adipose tissue appearing largely normal. One-week post-irradiation, a reduction in bone marrow tissue proliferation was observed, characterized by a decrease in hematopoietic cells and an increase in adipose tissue. Histological analysis of the right FN and L7 marrow revealed an increase in the proportion of fat vacuole area at 1-week post-radiotherapy, with the proportion of adipocyte vacuole area peaking increase compared to baseline, followed by a gradual decline over subsequent weeks (Figures 5A–D). Despite this reduction, fat vacuole area remained significantly elevated compared to non-irradiated controls at all timepoints (1−4 weeks post-irradiation, *P* < 0.001).

The Ca content measurements in right FN and L7 demonstrated a decline at different radiotherapy intervals, aligning with the Ca quantification derived from spectral CT and serum (Figures 5E–G). A strong linear correlation was observed between true concentrations at right FN by Ca content detection and Ca (Fat) value measured by DECT (r^2^ = 0.949, *P* < 0.01) (Figure 5H).

## 4 Discussion

The present study leveraged DECT imaging to longitudinally characterize the dynamic changes in Ca, HAP, and fat content within the L7 vertebral body and bilateral FNs during the critical early phase (1−4 weeks) post-irradiation. Crucially, these quantitative CT-derived measurements were compared with established histological and thermogravimetric analysis performed on the same tissue specimens. Our study revealed novel findings: (i) A decline pattern of Ca and HAP concentrations in L7 vertebrae and FNs across post-radiotherapy intervals, characterized by an initial slow decrease, followed by accelerated reduction, and subsequent deceleration; (ii) A fat content pattern demonstrating initial elevation followed by gradual decline with prolonged radiation exposure intervals. The findings detail the sequential alterations in these key bone constituents and demonstrate the potential utility of DECT as a non-invasive tool for monitoring radiation-induced changes in bone composition. To the best of our knowledge, previous investigations of radiation-related osseous damage have predominantly focused on long bone diaphyses or metaphyses, with limited targeted analyses of spinal vertebrae and FN regions (23).

While murine models remain the predominant choice for investigating radiation-induced bone pathology, we selected rabbits for this study due to their superior physiological dynamism and structural resemblance to humans, thereby enhancing clinical translatability (24). In addition, the larger body size of rabbits compared to rodents (e.g., mice or rats) facilitates surgical manipulations. The 30 Gy radiation dose utilized in this study was chosen according to previous findings indicating that a single high-dose exposure causes skeletal damage, thus enabling the evaluation of bone alterations induced by radiation (25, 26). Yamamoto et al. pointed out that the lumbar fracture rate after radiotherapy for cervical cancer could reach 29.8% (27). Another study shows that the majority of fractures (90%) that occur in elderly women after pelvic radiotherapy are hip fractures (including FN fractures), and the probability of nonunion and avascular necrosis of the femoral head after FN fractures is extremely high. It may also lead to the occurrence of various complications, and in severe cases, it can even endanger life (9, 28). Given the elevated fracture risk observed in the L7 vertebrae and femoral neck (FN) compared with other skeletal sites following radiotherapy, and considering the particular vulnerability of trabecular-rich regions to radiation-induced bone loss (9, 24), the L7 vertebrae and FN were selected for BMD measurements in this study.

Although conventional CT can assess bone mineral density (BMD) via CT values, it fails to delineate whether reduced BMD stems from decreased bone mineral content or increased adipose infiltration. In contrast, DECT utilizes material decomposition techniques to quantitatively discriminate changes in mineral and lipid components. In our phantom study, DECT demonstrated excellent accuracy in quantifying Ca concentration (*R*^2^ = 0.995), validating its reliability for BMD assessment, which further corroborates previous findings (19). Guided by the reported correlations between HAP/Ca density and QCT-BMD (29, 30) and the potential of MRI-measured fat content to reflect bone status (31), we selected HAP, Ca, and fat to form base material pairs.

In this study, we observed a progressive decline in bone Ca and HAP content following radiotherapy compared to control groups, and it showed a pattern of a slow decline at first, then a rapid decline, and then a slow decline again. Furthermore, muffle furnace assays confirmed reduced calcium levels post-irradiation and demonstrated a strong correlation with DECT-derived calcium parameters (*r*^2^ = 0.949, *P* < 0.01), validating the reliability of our DECT measurements. This finding is consistent with the results reported by Thio et al., who observed an immediate reduction in volumetric bone mineral density (vBMD) in irradiated vertebrae compared to non-irradiated controls (*P* = 0.010) using quantitative CT analysis (32). Another study in mice has shown fractionated abdominal irradiation (3 Gy × 2 per day × 7.5 days) induced BMD loss 10 days after the last fraction: 13.8% in the femur, and the loss of BMD in the femurs progressed with time (33). In our study, by week 2 post-radiotherapy, bilateral FNs exhibited similar declines in mineral content (left: Ca−14.5%, HAP−14.3%; right: −16.0 and −14.8%, all *P* < 0.001 vs. baseline). A comparable reduction was observed in the L7 vertebra (−17.8% Ca, −17.3% HAP, all *P* < 0.001 vs. baseline), consistent with the FN. Since ionizing radiation directly damages osteoblasts and osteogenic cells while stimulating osteoclast activity via upregulation of pro-resorptive cytokines (e.g., RANKL) (34), and both L7 and the FN are rich in trabecular bone, their parallel decline is mechanistically plausible. Wright et al. demonstrated through quantitative micro-CT analysis that significant trabecular deterioration—characterized by reduced bone volume fraction (BV/TV) and trabecular number (Tb.N), coupled with increased trabecular separation (Tb.Sp)—occurs in the FN within 7 days post-irradiation compared to sham controls (35). These microarchitectural alterations show remarkable concordance with our DECT findings of progressive Ca/HAP depletion and marrow adiposity expansion.

We speculate that the possible reasons of the trend are as follows: (i) immediate osteoblast dysfunction (0–1 week) due to direct cellular damage, (ii) subsequent osteoclast hyperactivation (1–3 weeks) mediated by pro-resorptive signaling, and (iii) eventual tissue remodeling (3–4 weeks) through progenitor cell recruitment (36, 37). Our findings at these different radiotherapy intervals indicate that the abscopal degradation of the skeleton after pelvic irradiation is not only a detrimental event that occurs early but also an injury that progresses with time (at least in the L7 and the FN after a single dose of radiation at 30 Gy). Such early Ca and HAP loss, detectable by DECT, may serve as a predictive biomarker for fracture risk.

Our DECT analysis reveals a characteristic biphasic response of BMAT to radiation: an initial peak at 1 week (42.3 ± 5.7% increase, *p* < 0.01) followed by progressive decline (8.2 ± 1.3%/week, *p* < 0.05). This “peak-and-decline” pattern, first documented here, parallels the histopathological changes and may involve two distinct mechanisms: (i) early BMAT expansion due to hematopoietic cell apoptosis/adipocyte differentiation as validated through high-resolution magic angle spinning proton nuclear magnetic resonance spectroscopy (HRMAS ^1^H-NMRS) (38) and DECT studies (*r* = 0.80 vs. histology) (39), and (ii) subsequent BMAT reduction potentially through adipocyte-osteoblast transdifferentiation (40, 41). The observed dynamics likely reflect combined microenvironmental remodeling (42) and perfusion-mediated changes (DCE-MRI evidence (38, 43)). These quantitative DECT measurements of BMAT kinetics offer novel biomarkers for optimizing bone marrow-sparing radiotherapy and predicting fracture risk.

Several constraints warrant acknowledgment in this investigation. Primarily, the restricted cohort size (*n* = 30 rabbits) coupled with a brief 4-week observation window may constrain the extrapolation of our results to long-term radiation-induced pathologies. Additionally, the absence of standardized adverse effect grading using the VRTOG v2.0 criteria limits the quantitative assessment of radiation-induced toxicity, and this should be recognized as another limitation of the present study; future investigations will adopt the VRTOG v2.0 to evaluate adverse reactions in animals, thereby strengthening the rigor of the assessment. Furthermore, inherent species-specific disparities necessitate prudent interpretation when translating these animal findings to human pathophysiology. Subsequent inquiries should leverage retrospective clinical archives to corroborate the documented longitudinal bone mineral density trajectories. Moreover, integrating dual-energy computed tomography with sophisticated magnetic resonance protocols, such as diffusion-weighted imaging, or circulating biomarkers including CTX and P1NP, could provide deeper mechanistic insights into radiation-induced bone injury.

## 5 Conclusion

DECT is a non-invasive imaging examination method. This study systematically elucidates the dynamic changes in Ca, HAP, and fat content in L7 vertebrae and FN following radiotherapy using DECT technology. Our research showed a decline pattern of Ca and HAP concentrations across post-radiotherapy intervals, characterized by an initial slow decrease, followed by accelerated reduction, and subsequent deceleration. Fat content reached its maximum in the first week, followed by a gradual decrease in the second week. These patterns seen on DECT in rabbit bone were consistent with histopathologic findings. They may be useful to detect early radiation-induced bone metabolic dysfunction. Its integration into radiotherapy planning and post-treatment monitoring could improve risk stratification for pelvic fractures, guide personalized interventions, and improve patients’ quality of life.

## Data Availability

The raw data supporting the conclusions of this article will be made available by the authors, without undue reservation.
